# Von Willebrand factor and ADAMTS13 activity in relation to risk of dementia: a population-based study

**DOI:** 10.1038/s41598-018-23865-7

**Published:** 2018-04-03

**Authors:** Frank J. Wolters, Johan Boender, Paul S. de Vries, Michelle A. Sonneveld, Peter J. Koudstaal, Moniek P. de Maat, Oscar H. Franco, M. Kamran Ikram, Frank W. Leebeek, M. Arfan Ikram

**Affiliations:** 1 Department of Epidemiology, Erasmus Medical Centre, Rotterdam, The Netherlands; 2 Department of Neurology, Erasmus Medical Centre, Rotterdam, The Netherlands; 3 Department of Haematology, Erasmus Medical Centre, Rotterdam, The Netherlands; 4 Human Genetics Center, Department of Epidemiology, Human Genetics, and Environmental Sciences, School of Public Health, The University of Texas Health Science Center at Houston, Houston, Texas USA

## Abstract

Low ADAMTS13 activity is associated with an increased risk of cardiovascular disease, which is generally attributed to its proteolytic effects on Von Willebrand factor (VWF). Cardiovascular health is an important determinant of cognitive decline, but the association of either VWF or ADAMTS13 with risk of dementia is unknown. Between 1997–2002, we measured VWF antigen and ADAMTS13 activity in 6055 participants of the population-based Rotterdam Study (mean age 69.3 years, 57.2% women). At baseline, 85 participants had dementia, and during 15 years of follow-up 821 developed dementia. Higher VWF was associated with prevalence and risk of dementia, unaffected by concurrent ADAMTS13 activity, but estimates strongly attenuated over time and were no longer statistically significant at 4 years of follow-up (relative risks [95% CI] per standard deviation increase– cross-sectional: 1.37 [1.06–1.77], and longitudinal: 1.05 [0.97–1.14]). In contrast, low ADAMTS13 was associated with increased risk of dementia throughout follow-up (hazard ratio per SD decrease– 1.16 [1.06–1.28]), which alike for ischaemic stroke, was modified by the presence of diabetes (*P*-interaction = 0.003). In conclusion, higher VWF and low ADAMTS13 activity are associated with increased risk of dementia, but differences in time-course and lack of synergistic effects may indicate in part independent underlying mechanisms.

## Introduction

Von Willebrand factor (VWF) is a large multimeric glycoprotein with critical functions in haemostasis. Deficiency or dysfunction of VWF, known as Von Willebrand disease, can cause prolonged or excessive bleeding^1^, whereas high levels of VWF antigen have been associated with increased risk of cardiovascular disease^2^. *In vivo* effects of VWF largely depend on the proteolytic activity of ADAMTS13 (A Disintegrin And Metalloproteinase with a ThromboSpondin type 1 motif, member 13). ADAMTS13 cleaves large, haemostatically highly reactive VWF multimers into smaller, less active multimers. Consequently, high VWF may lead to a hypercoagulable state in particular when ADAMTS13 activity is low, and a combined measure of VWF and ADAMTS13 could thus more accurately capture the biological activity of VWF^3^. We have previously shown that low activity of ADAMTS13 itself is associated with increased risk of cardiovascular disease^4–8^, while the combination of VWF and ADAMTS13 appears indeed more strongly associated with stroke risk than what would be expected on the basis of the individual measurements^5^.

Vascular disease and thrombosis play an important role in the aetiology of dementia, including Alzheimer’s disease^9^. Accordingly, a recent meta-analysis of cross-sectional studies concluded that VWF antigen levels are higher in patients with dementia than in controls^10^. However, of two studies that assessed the risk of dementia by VWF^11,12^, neither found baseline VWF antigen levels associated with dementia risk after 4 and 17 years of follow-up, respectively, albeit the latter was hampered by substantial attrition (50%) and lack of cognitive screening at baseline. Apart from methodological considerations, release of VWF from damaged endothelial cells in later stages of cognitive impairment may explain why profound cross-sectional associations do not extend to longer term follow-up. However, the time-course of the association between VWF and dementia remains unknown, and although ADAMTS13 could aid in disentangling haemostatic effects from associations marking endothelial damage, no published studies about VWF and dementia took into account concurrent ADAMTS13 activity.

While VWF is the only known substrate for ADAMTS13, several studies suggest that ADAMTS13 might have functions beyond VWF cleavage. Suggested roles include inflammation, angiogenesis, and extracellular matrix integrity^13^, each of which have been implicated also in the aetiology of dementia^14–16^. A versatile role of ADAMTS13 was further suggested, when we recently showed that *high* activity of ADAMTS13 relates to a *higher* risk of diabetes in the general population^17^. The underlying mechanisms remain elusive, but these studies jointly highlight the need for investigation of ADAMTS13 in the context of, as well as beyond its proteolytic activity of VWF.

We aimed to determine the cross-sectional and long-term associations of VWF and ADAMTS13 with cognitive decline and dementia risk in a population-based study. We investigated independent and synergistic effects of VWF and ADAMTS13, and explored these associations in the context of prior studies linking ADAMTS13 to diabetes, angiogenesis, and extracellular matrix integrity.

## Methods

This study is part of the Rotterdam Study, a large ongoing population-based cohort study in the Netherlands, with an initial study population of 7,983 participants aged ≥ 55 years from the Ommoord area, a suburb of Rotterdam. In 2000, the cohort was expanded with an additional 3011 participants who moved into the study area or reached age 55. The Rotterdam Study methods have been described previously^18^. Briefly, participants were interviewed at home and subsequently examined at the research centre for baseline assessment from 1990 to 1993 (baseline cohort) and 2000 to 2002 (expansion cohort), with follow-up examinations every 4 years. Citrated plasma samples were collected at the third visit of the original cohort (1997–1999), and the first visit of the expansion cohort (2000–2002), which are the baseline of the current study. Of 9,030 surviving participants at the time, 7,510 participated in this examination cycle, of whom 6,735 visited the study centre. Of these, 43 had insufficient cognitive screening to determine dementia status.

### Ethical approval and data availability

The Rotterdam Study has been approved by the medical ethics committee according to the Population Study Act Rotterdam Study, executed by the Ministry of Health, Welfare and Sports of the Netherlands. Written informed consent was obtained from all participants. Rotterdam Study data can be made available to interested researchers upon request. Requests can be directed to data manager Frank J.A. van Rooij (f.vanrooij@erasmusmc.nl). We are unable to place data in a public repository due to legal and ethical restraints. Sharing of individual participant data was not included in the informed consent of the study, and there is potential risk of revealing participants’ identities as it is not possible to completely anonymize the data. This is of particular concern given the sensitive personal nature of much of the data collected as part of the Rotterdam Study.

### Measurement of Von Willebrand factor antigen and ADAMTS13 activity

Fasting venous blood samples were taken at the research centre, and citrated plasma was stored at –80 °C. We determined VWF antigen with an in-house enzyme-linked immunosorbent assay using polyclonal rabbit antihuman VWF antibodies (DakoCytomation, Glostrop, Denmark) for catching and tagging. The intra-assay coefficient of variation was 5.8% and the inter-assay coefficient of variation was 7.8%. We measured ADAMTS13 activity using a kinetic assay based on the fluorescence resonance energy transfer substrate VWF73 (FRETSVWF73) assay^19^. This assay uses a peptide containing the ADAMTS13 cleavage site of VWF, and thus captures variation in the VWF cleavage rate determined by ADAMTS13 levels and structure. Plasma samples were measured against a reference curve of serial dilutions of normal human plasma defined to have an ADAMTS13 activity of 1 IU/mL, and we expressed ADAMTS13 activity as a percentage of this. Ten percent of the samples were retested and all were within 25% variation. From these measurements, we also calculated the ratio between ADAMTS13 activity and VWF antigen levels.

### Cognitive function assessment and dementia screening

Participants underwent detailed tests to determine cognitive function, comprising the Stroop test (error adjusted time in seconds taken for completing a reading/colour naming interference task), the letter-digit substitution task (number of correct digits in 1 minute), and the verbal fluency test (number of animal species within 1 minute)^20^. Cognitive function was assessed at baseline (i.e. time of blood sampling) and at three subsequent follow-up examinations (after a mean follow-up of 4.4 (SD 0.6), 10.8 (SD 0.6), and 15.4 (SD 0.7) years, respectively). To obtain a composite measure of test performance, we calculated the G-factor, which explained approximately 61% of variance in cognitive test scores at each examination round in our population. For each participant, Z-scores were calculated for each test separately, by dividing the difference between individual test score and population mean by the population standard deviation.

Participants were screened for dementia at baseline and subsequent centre visits^21^. Those with a Mini-Mental State Examination score < 26 or Geriatric Mental Schedule score > 0 underwent further investigation and informant interview, including the Cambridge Examination for Mental Disorders of the Elderly. In addition, the entire cohort was under continuous surveillance for dementia through electronic linkage of the study database with medical records from general practitioners and the regional institute for outpatient mental health care. A consensus panel led by a consultant neurologist established the final diagnosis according to standard criteria for dementia (DSM-III-R) and Alzheimer’s disease (NINCDS–ADRDA). Within this period, participants were censored at date of dementia diagnosis, death, loss to follow-up, or 1^st^ January 2015, whichever came first.

### Other measurements

We assessed smoking status (i.e. current, former, never) and use of antihypertensive, lipid-lowering, glucose lowering, and antithrombotic (i.e. coumarine derivatives or platelet inhibitors) medication at baseline by interview. Systolic and diastolic blood pressures were measured with a random-zero sphygmomanometer. Fasting serum lipid levels, C-reactive protein (CRP) and fibrinogen were measured at baseline. Diabetes, prediabetes and normoglycaemia were defined according to WHO guidelines^22^. *APOE* genotype was determined using polymerase chain reaction on coded DNA samples (baseline cohort), and using a bi-allelic TaqMan assay (rs7412 and rs429358; expansion cohort). ABO blood group antigen phenotypes were reconstructed by haplotype analysis of 3 single nucleotide polymorphisms (rs8176749, rs8176704, and rs505922), and classified into O and non-O. We determined history of stroke and myocardial infarction by interview, consultation of medical records, and abnormalities on electrocardiography.

### Measurement of other blood markers

In a subset of 1075 non-demented participants, we measured at baseline 150 plasma markers via multiplex immunoassay on human multianalyte profiles in the fasting blood samples collected at baseline (Myriad RBM Inc., Austin TX, USA; http://rbm.myriad.com). Of these, we selected markers with an identified role in angiogenesis (i.e. angiopoietin-2 (ANG-2), vascular endothelial growth factor (VEGF), platelet-derived growth factor (PDGF), transforming growth factors α and β (TGF-α, TGF-β)) or related to the extracellular matrix (i.e. matrix metalloproteinases MMP-2, MMP-3, and MMP-9, tissue inhibitor of metalloproteinase-1 (TIMP-1), Tenascin-C, connective tissue growth factor (CTGF)), based on suggested roles of ADAMTS13 beyond regulation of thrombosis^13^. The assay did not pass quality control (>20% unmeasurable) for TGF-α, TGF-β, MMP-2, MMP-9 and CTGF, leaving 6 markers for analysis (all with measurements in ≥92.4% of participants).

### Analysis

Because of a right-skewed distribution of VWF and the ADAMTS13:VWF-ratio, we performed a natural logarithmic transformation to obtain a roughly normal distribution of the data. We computed Z-scores for each individual by dividing the difference between the individual value and the population mean by the population standard deviation.

Missing covariate data (15.0% for ABO blood type, and <5.0% for all other covariables) were imputed using 5-fold multiple imputation. Distribution of covariates was similar in the imputed versus non-imputed dataset. All analyses were adjusted for age, sex, and study subcohort. In a second model we further adjusted for systolic and diastolic blood pressure, use of antihypertensive medication, serum total cholesterol, high-density lipoprotein (HDL) cholesterol and triglycerides, use of lipid-lowering medication, body mass index, diabetes mellitus, creatinine, CRP, fibrinogen, ABO blood type, and use of antithrombotic medication.

We determined the association of VWF and ADAMTS13 with prevalence and incidence of dementia, using logistic regression and Cox proportional hazard models, respectively. As the proportional hazard assumption was violated for VWF, we also determined associations with dementia risk per year increase in follow-up. We determined risk of dementia per standard deviation (SD) increase as well as per quartile of VWF, ADAMTS13, and their ratio. In view of previously suggested threshold effects of ADAMTS13, we also compared the lowest quartile of ADAMTS13 to the highest three quartiles altogether^2^. We assessed effect modification by (pre-)diabetes, by testing for multiplicative interaction in the fully adjusted Cox model. We repeated the analysis after excluding participants with prevalent myocardial infarction or stroke, while censoring at time of incident myocardial infarction or stroke in the fully adjusted model. We performed further sensitivity analyses, 1) for Alzheimer’s disease only, 2) stratifying by the mean age of the study population (i.e. 69.3 years), 4) stratifying by sex, and 5) stratifying by blood type O versus non-O.

We then determined the association of VWF levels and ADAMTS13 activity with change in scores on cognitive assessment during follow-up, using linear mixed models. We fitted a model (restricted maximum likelihood) to the G-factor of cognitive scores, including age, sex, follow-up time, time*age, VWF/ADAMTS13, and time*VWF/ADAMTS13 in the model. We chose a diagonal covariance structure (heterogeneous variance and zero correlation between elements) for the random effects, including a random intercept and follow-up time, and added other covariates in agreement with the fully adjusted model described above. We repeated the analysis for all cognitive tests, stratified by diabetic status, and limited to the 1^st^, 2^nd^, and 3^rd^ follow-up examination, respectively.

Finally, in the subset of participants with immunoassay data, we determined correlations of ADAMTS13 with ANG-2, VEGF, PDGF, MMP-3, TIMP-1, and Tenascin-C, using linear regression (of natural log-transformed values if so required to obtain normal distributions of the data). Values exceeding ± 3.5 standard deviations from the mean were excluded from analysis. We fitted univariable models, and additional models including age, sex, and each of the other biomarkers, whilst applying the Benjamini-Hochberg correction for multiple testing.

All analyses were done using SPSS Statistics 21.0 (IBM Corp, Armonk, NY, USA) or R statistical software 3.1.1 (package ‘nlme’). Alpha level was set at 0.05.

## Results

Among 6,692 eligible participants, we could not determine VWF antigen in 380 participants and ADAMTS13 activity in 628 participants, mainly due to technical reasons or insufficient blood sampling, leaving 6055 (90.5%) participants with both measures for analyses. Baseline characteristics of the study population are presented in Table 1.

**Table 1:** Baseline characteristics of the 6,055 participants.

Age (years)	69.3 (±8.2)
Female sex	3,461 (57.2)
Systolic blood pressure (mmHg)	143 (±21)
Diastolic blood pressure (mmHg)	77 (±11)
Antihypertensive medication	2,017 (35.0)
Pre-diabetes	1,663 (28.1)
Diabetes	744 (12.6)
Serum cholesterol (mmol/L)	5.82 (±0.98)
Serum HDL cholesterol (mmol/L)	1.39 (±0.39)
Serum triglycerides (mmol/L; median, IQR)	1.35 (1.03–1.81)
Lipid-lowering medication	746 (12.8)
Smoking	
Former	2,958 (49.3)
Current	1,032 (17.2)
Creatinine (mg/dL)	0.89 (±0.21)
Body-mass index (kg/m^2^)	26.9 (±4.0)
History of cardiovascular disease	283 (4.7)
Anti-thrombotic medication	1,135 (18.7)
*APOE* genotype	
3/3	3,389 (58.1)
ε2/2 or ε2/3	821 (14.1)
ε2/4 or ε3/4, ε4/4	1,627 (27.9)
Von Willebrand factor (IU/mL; median, IQR)	1.20 (0.93–1.60)
ADAMTS13 (%)	91.5 (±17.7)
Fibrinogen (g/L; median, IQR)	3.8 (3.3–4.4)
C-reactive protein (mg/mL; median, IQR)	1.8 (0.7–3.7)
Blood type O	2348 (45.6)

Data are presented as frequency (%) for categorical, and mean ± standard deviation for continuous variables, unless indicated otherwise; IQR = interquartile range.

### Prevalent dementia

At baseline, 85 participants had dementia, of whom 68 had Alzheimer’s disease. Participants with dementia had higher VWF antigen levels and lower ADAMTS13 activity than individuals without dementia (Table 2). Consequently, the ADAMTS13:VWF ratio was also lower in individuals with dementia, although adjustment for ADAMTS13 did not change the estimates for VWF, and ADAMTS13 did not modify the association of VWF with dementia (Table 2; *P*-interaction = 0.93). Associations of VWF and ADAMTS13 with dementia were mildly attenuated for Alzheimer’s disease only, and broadly unaltered by excluding participants with cardiovascular disease (Supplementary Table S1).

**Table 2:** Von Willebrand factor (VWF) and ADAMTS13 at baseline in relation to the prevalence of dementia.

	All-cause dementia (Model I)		P-value	All-cause dementia (Model II)		P-value
	No (n = 5,970)	Yes (n = 85)		No (n = 5,970)	Yes (n = 85)	
**VWF:Ag**						
Geometric mean (95% CI)	1.22 (1.20–1.23)	1.34 (1.23–1.46)	0.021	1.23 (1.20–1.26)	1.36 (1.26–1.48)	0.013
OR (95% CI) per SD increase	1.29 (1.04–1.62)		0.023	1.37 (1.06–1.77)		0.017
**ADAMTS13 activity**						
Mean (95% CI)	91.2 (90.7–91.6)	86.1 (82.5–89.7)	0.007	91.2 (90.1–92.4)	86.9 (83.2–90.6)	0.015
OR (95% CI) per SD decrease	1.28 (1.01–1.64)		0.046	1.25 (0.95–1.63)		0.107
**ADAMTS13:VWF ratio**						
Geometric mean (95% CI)	73.5 (72.6–74.3)	61.9 (56.3–68.2)	0.001	72.5 (70.5–74.7)	61.6 (56.1–67.6)	0.0004
OR (95% CI) per SD decrease	1.39 (1.12–1.72)		0.003	1.44 (1.13–1.85)		0.004

n = number of participants; SD = standard deviation; OR = odds ratio from logistic regression model; CI = confidence interval. Geometric means facilitate a comparison of normalized results, as is the case for the not normally distributed VWF and the ADAMTS13:VWF ratio.

Model I: adjusted for age, sex, study subcohort.

Model II: model I with additional adjustment for smoking, systolic and diastolic blood pressure, antihypertensive medication, diabetes, serum cholesterol, high density lipoprotein cholesterol and triglycerides, lipid-lowering medication, body mass index, creatinine, antithrombotic medication, fibrinogen, C-reactive protein, ABO blood type, and *APOE* genotype.

### Longitudinal assessment

Of 5,970 non-demented participants at baseline, 821 participants were diagnosed with dementia during a mean follow-up of 11.6 years (follow-up was complete for 97.5% of potential person years). Of all dementia diagnoses, 671 were due to Alzheimer’s disease, and 154 were preceded by myocardial infarction or a stroke. At baseline, 5,844/5,970 (97.9%) participants underwent extensive cognitive assessment, of whom 4,582 (78.4%) underwent at least 2 assessments, and 2,934 (50.2%) attended at least three examinations.

### VWF and cognitive decline/ incident dementia

**Figure 1:**
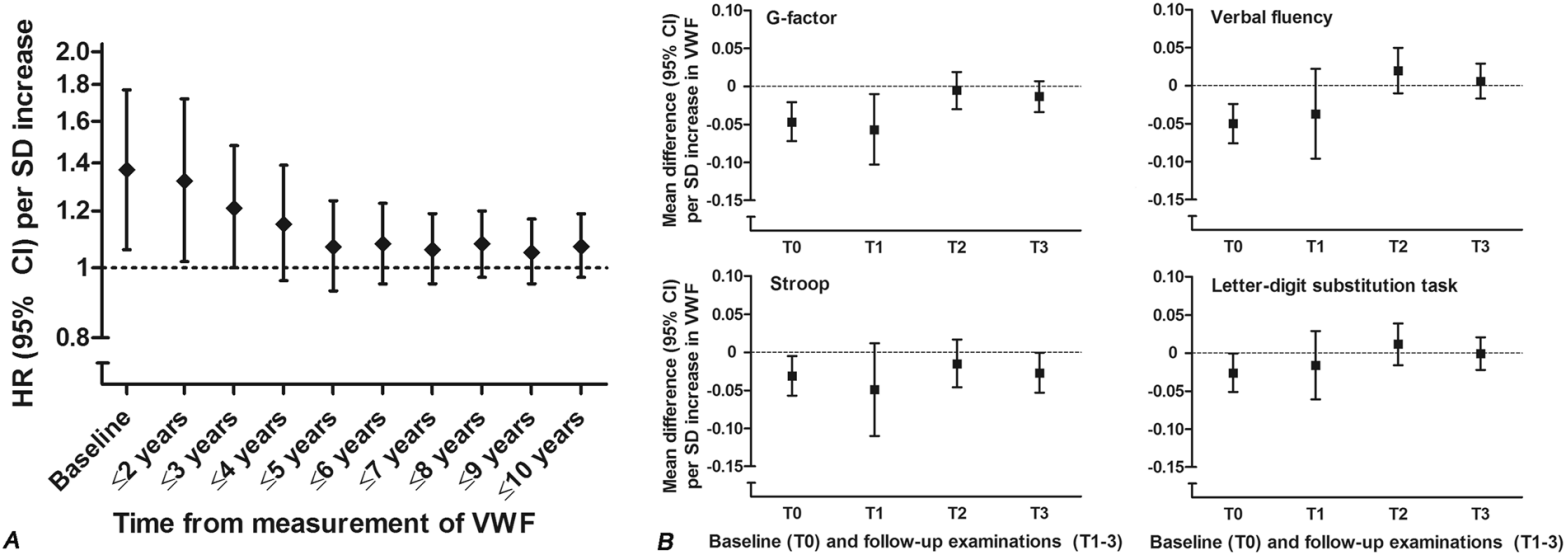
Von Willebrand factor (VWF) in relation to risk of dementia and cognitive performance over time. (**A**) Cross-sectional estimates at baseline (odds ratio from logistic regression) are followed by hazard ratios for the risk of incident dementia in longitudinal analyses including one extra year of follow-up from baseline per presented figure (Cox regression). Results are from the fully adjusted model. HR = hazard ratio; CI = confidence interval; SD = standard deviation. (**B**) Results reflect the betas per standard deviation increase for baseline VWF and the VWF*follow-up time interaction (expressed per 10 years follow-up) from a fully adjusted linear mixed model including all four examinations, and restricting analyses to the first two or three assessments, respectively. T0 = baseline; T1 = first follow-up examination after 4.4 years; T2 = second follow-up examination after 10.8 years; T3 = third follow-up examination after 15.4 years. Lower scores reflect worse performance for all tests. Presented cross-sectional estimates from the model including all examinations were robust in the time-restricted models.

Overall, VWF antigen levels were not associated with risk of dementia (adjusted HR per SD increase: 1.05, 0.97–1.14). VWF levels were, however, associated with short-term risk of dementia, but these associations attenuated over time and were no longer statistically significant beyond 4 years of follow-up (Fig. 1A; for a full table see Supplementary Table S2). Similarly, associations of VWF with cognitive test performance at baseline extended to the first re-examination at 4.4 years, but not thereafter (Fig. 1B; for results per quartile of VWF see Supplementary Table S3). The associations of VWF with cognitive decline and risk of dementia were not affected by concurrent ADAMTS13 activity (*P*-value for the interaction VWF*ADAMTS13 = 0.58 for all-cause dementia and and 0.85 the G-factor).

### ADAMTS13 and cognitive decline/ incident dementia

Low ADAMTS13 activity was associated with an increased risk of dementia (Table 3), with similar effect estimates throughout follow-up. The association was modified by the presence of impaired fasting glucose or diabetes (*P*-interaction = 0.003), such that low activity of ADAMTS13 related to higher risk of dementia primarily in non-diabetics, but not in those with (pre-)diabetes (Table 3). This opposite direction of effect was similar for impaired fasting glucose and diabetes (Supplementary Table S4), and unaffected by excluding individuals using antidiabetic medication. Risk estimates of ADAMTS13 itself were consistently stronger than those of the ADAMTS13:VWF ratio (Supplementary Table S5), and in contrast to ADAMTS13 there was no interaction between (pre-)diabetes and VWF on the risk of dementia (*P*-value for the VWF*(pre-)diabetes interaction = 0.99).

**Table 3:** Baseline ADAMTS13 in relation to the risk of dementia in the overall population, and stratified by (pre-)diabetic status.

ADAMTS13 activity	Overall study population		Free of (pre-)diabetes		With (pre-)diabetes	
	n_dem_/N_tot_	HR (95% CI)	n_dem_/N_tot_	HR (95% CI)	n_dem_/N_tot_	HR (95% CI)
Per quartile						
Q1_<80.6%_	239/1,492	1.16 (0.94–1.43)	190/1081	1.51 (1.16–1.95)	49/401	0.64 (0.43–0.95)
Q2_80.6–91.2%_	210/1,493	1.00 (0.82–1.23)	158/1088	1.22 (0.94–1.57)	51/389	0.67 (0.46–0.97)
Q3_91.2–101.9%_	191/1,493	0.85 (0.69–1.04)	133/1095	0.90 (0.69–1.17)	58/391	0.77 (0.54–1.10)
Q4_>101.9%_	181/1,492	reference	106/995	reference	74/482	reference
Q1 versus Q2–4		1.23 (1.05–1.44)		1.44 (1.20–1.73)		0.81 (0.58–1.13)
Per SD decrease	821/5,970	1.06 (0.98–1.15)	587/4259	1.16 (1.06–1.28)	232/1663	0.90 (0.79–1.03)

HR = hazard ratio from Cox proportional hazard regression; CI = confidence interval; n_dem_ = number of dementia cases and N_tot_ = number of individuals in group, presented for non-imputed data (missing diabetes status, n = 48).

Model adjusted for age, sex, study subcohort, smoking, systolic and diastolic blood pressure, antihypertensive medication, serum cholesterol, HDL cholesterol and triglycerides, lipid-lowering medication, body mass index, (pre-)diabetes (if applicable), creatinine, antithrombotic medication, CRP, fibrinogen, and *APOE* genotype.

**Figure 2:**
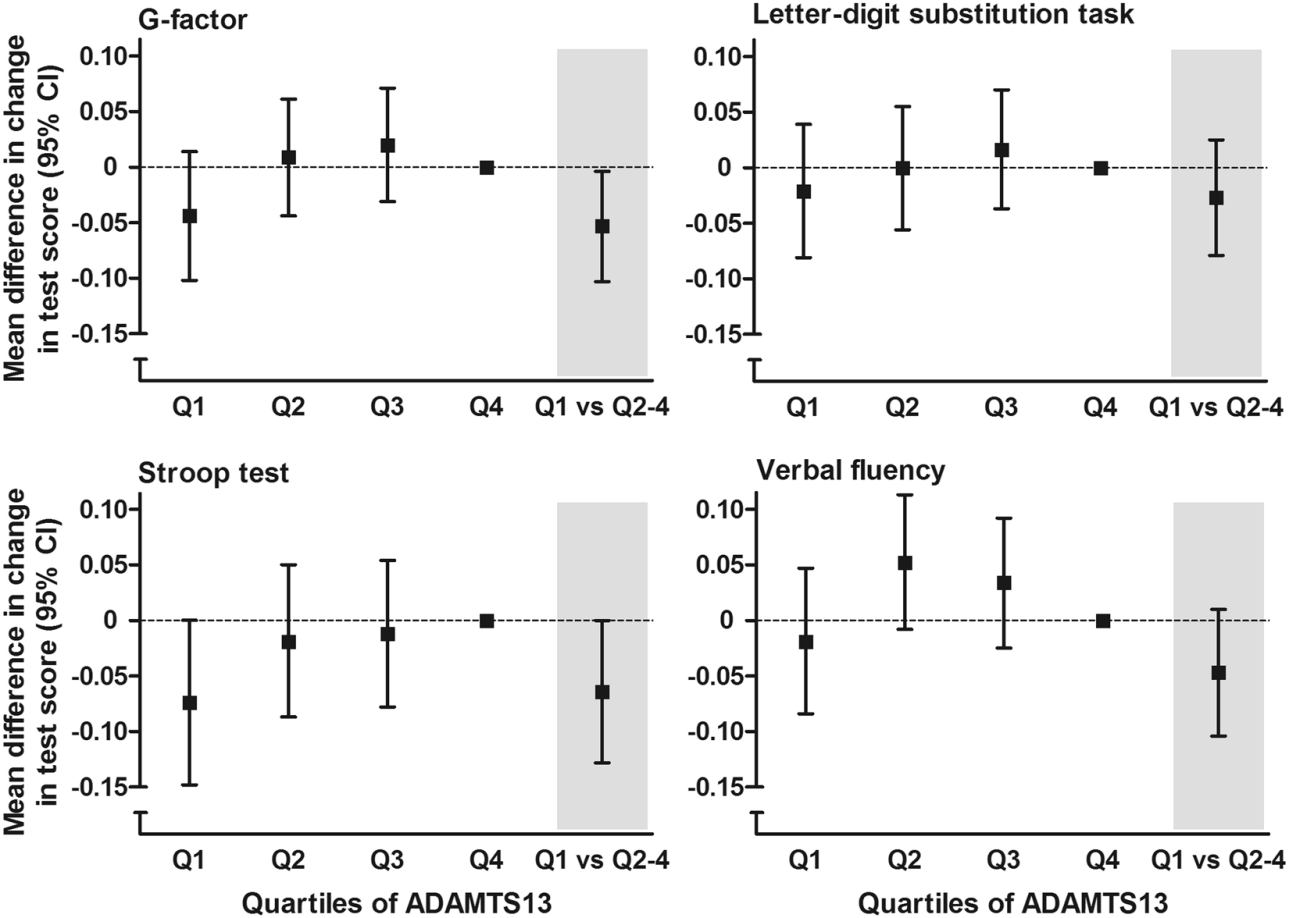
ADAMTS13 activity and change in cognitive test scores during four consecutive examination rounds. Change in cognitive performance per 10 years of follow-up, expressed per quartile of ADAMTS13 relative to the highest quartile, and comparing low versus normal ADAMTS13 activity. Lower scores reflect worse performance for all tests. Model adjusted for age, sex, smoking, systolic and diastolic blood pressure, antihypertensive medication, serum cholesterol, HDL cholesterol and triglycerides, lipid-lowering medication, body mass index, diabetes, creatinine, antithrombotic medication, CRP, fibrinogen, ABO blood type, and *APOE* genotype.

ADAMTS13 was associated with more rapid decline in cognitive test performance during 15 years of follow-up (Fig. 2), which was again most profound in individuals without diabetes (Supplementary Table S6). The ADAMTS13:VWF ratio was also associated with change in cognitive test performance, with similar effect estimates, except for a somewhat stronger association with performance on the Stroop test (Supplementary Table S7). Associations were broadly unaltered after excluding all participants who developed dementia during follow-up (Supplementary Table S8).

### Sensitivity analyses

In further sensitivity analyses, associations of VWF and ADAMTS13 with dementia were similar for Alzheimer’s disease only, and unaffected by excluding those with prevalent cardiovascular disease and censoring participants at time of myocardial infarction or stroke during follow-up (Supplementary Figure S1). We found no evidence of effect modification by age at blood sampling, sex, or ABO blood type (Supplementary Figure S1; all *P*-values for interaction ≥ 0.15).

### ADAMTS13 and immunoassay markers

Among a random subset of 1075 participants with immunoassay biomarker measurements, lower ADAMTS13 activity was significantly associated with higher levels of VEGF, MMP-3, and Tenascin-C, but not ANG-2 and PDGF (Table 4). The univariable association between MMP-3 and ADAMTS13 attenuated after adjustment for age and sex, but differed substantially by concurrent levels of TIMP-1 (*P*-value for interaction = 0.01), such that associations were strongest in the presence of high TIMP-1. A similar interaction was seen between Tenascin-C and TIMP-1 (*P*-value for interaction = 0.05).

**Table 4:** ADAMTS13 and selected markers of angiogenesis and extracellular matrix integrity.

	Model I β, 95% CI	Model II β, 95% CI	Model II – low TIMP-1^†^ β, 95% CI	Model II – high TIMP-1^†^ β, 95% CI
ANG-2	0.01 (−1.25; 1.27)	0.72 (−0.62; 2.06)	n/a	n/a
PDGF	0.64 (−0.41; 1.68)	0.14 (−0.90; 1.19)	n/a	n/a
VEGF	−1.66 (−3.07; −0.25)^*^	−1.83 (−3.33; −0.34)^*^	n/a	n/a
MMP-3	−4.03 (−5.05; −3.00)^*†^	−1.37 (−2.64; −0.10)^†^	0.31 (−1.55; 2.17)	−2.93 (−4.69; −1.17)^*^
Tenascin-C	−1.74 (−2.78; −0.70)^*†^	−1.42 (−2.46; −0.37)^*†^	−0.68 (−2.16; 0.81)	−2.09 (−3.57; −0.61)^*^
TIMP-1	−1.31 (−2.47; −0.14)^*†^	1.40 (0.11; 2.69)^†^	n/a	n/a

ANG-2 = angiopoietin-2; PDGF = platelet-derived growth factor; VEGF = vascular endothelial growth factor; MMP-3 = matrix metalloproteinase-3; TIMP-1 = tissue inhibitor of metalloproteinases-1. Values represent change in ADAMTS13 activity per standard deviation increase in the specific marker.

Model I: univariable linear regression.

Model II: linear regression adjusted for age, sex, and all other biomarkers.

*Statistically significant at 0.05 level after correction for multiple testing.

^†^The univariable association between MMP-3 and ADAMTS13 largely attenuated after adjustment for age and sex. There was interaction of MMP-3 and Tenascin-C with TIMP-1 on the association with ADAMTS13 (p-values for interaction of 0.01 and 0.05, respectively, in the adjusted model); these associations are consequently presented separately for low (<median) and high (≥median) TIMP-1.

## Discussion

In this large population-based study, we found that higher VWF antigen levels are associated with prevalence and short-term, but not long-term risk of dementia. Low ADAMTS13 activity is associated with dementia risk during prolonged follow-up, with data suggesting an interactive mechanisms between ADAMTS13 and diabetes in the development of dementia. We did not observe synergistic effects of VWF and ADAMTS13 activity, which might indeed indicate in part independent underlying mechanisms.

The cross-sectional association between VWF and dementia in our study is in line with a recent meta-analysis, which reported similar (standardised) differences in VWF levels between individuals with all-cause dementia and controls^10^. However, we found associations to rapidly attenuate over the first few years of follow-up, explaining why two prior longitudinal studies did not find a significant association between VWF and risk of dementia after 4 and 17 years of follow-up, respectively^11,12^. The crucial role of time in this association could indicate high variability in VWF levels, either physiologically or induced by disease processes or treatment. Levels of VWF in the bloodstream may increase exponentially during the course of disease due to increasing severity of endothelial injury, and the biological effect of VWF may also vary with physiological changes in advanced stages of disease, such as wall shear stress^23,24^. This physiological variability could be investigated in future studies by incorporation of multiple measurements of VWF over time, which will prove important to determine to which extent prior associations of VWF with (subclinical) disease in fact reflect physiological activity of VWF, or are due to endothelial injury.

VWF has been associated with markers of cerebral small-vessel disease that are known risk factors for dementia^25,26^, including white matter hyperintensities on MRI and microhaemorrhages co-localised with beta-amyloid deposits^27,28^. As high VWF increases the risk of ischaemic stroke^29^, cerebral ischaemia could further link VWF to cognitive decline via (covert) brain infarcts or cortical micro-infarcts.

Such effects might be reduced in individuals with blood type O^30^, due to accelerated clearance and thus 25% lower levels of VWF^31,32^, although we did not find differential effects of VWF across blood type in our study. Beyond its direct effects, the function of VWF as a carrier protein for coagulation factor VIII (FVIII), thereby prolonging its half-life 10-fold^33^, might in part explain recently reported cognitive impairment with higher FVIII^30^. Finally, *in vitro* study suggests that inflammatory cytokines increase release and inhibit cleavage of VWF^34^, which might link inflammatory and ischaemic pathways in the pathogenesis of Alzheimer’s disease^14^. Future studies linking measurements of VWF to (longitudinal) magnetic resonance neuroimaging may further unravel these potential mechanisms.

In contrast to findings for VWF antigen, low ADAMTS13 activity was associated with cognitive decline and dementia risk throughout the 15-year follow-up in individuals without (pre-)diabetes. In line with reports of myocardial infarction and ischaemic stroke^5,8^, we observed increased risks only in the lower range of ADAMTS13, supporting a threshold effect in ADAMTS13 activity^2^. Nevertheless, the lower range of activity in the community is generally sufficient to maintain the equilibrium of VWF multimer formation and degradation^35,36^. Along with the effect estimates for ADAMTS13 generally exceeding those of the ADAMTS13:VWF ratio, this renders it unlikely that proteolytic effects of ADAMTS13 on VWF alone are accountable for the association of ADAMTS13 with dementia. Yet, most studies about ADAMTS13 have focused on its relationship with VWF or role in thrombotic thrombocytopenic purpura, and limited data are available to corroborate other pathways. Preliminary evidence suggests a role of ADAMTS13 in (downregulation of) inflammation^13,37^, regulation of angiogenesis^13^, and degradation of extracellular matrix^13^, which have also been described in dementia^14–16^. In mice, deficiency of ADAMTS13 enhances inflammation and plaque formation^38,39^, aggravates consequences of cerebral ischaemia^40–42^, and appears to regulate blood-brain barrier permeability^43^, possibly by controlling vascular remodeling via VEGF, ANG-2, and galectin-3 related pathways^42,43^. While these processes in mice often appear dependent on VWF or are observed in ADAMTS-/- mice, the levels required may be limited, and thus generally abundant in the general population. In exploratory analyses, we found associations of ADAMTS13 activity with levels of VEGF, MMP-3, Tenascin-C, and TIMP-1, which might indeed indicate involvement in vascular remodelling, and in any case encourage further study of ADAMTS13 in relation to vascular (brain) disease and neurodegeneration.

Our findings suggest that diabetes pathophysiology, rather than antidiabetic medication, modifies the association between ADAMTS13 and dementia risk. These analyses were prompted by our recent study in which we found increased risks of diabetes with higher ADAMTS13 activity^17^. Although the mechanisms underlying these observations are unknown, it is conceivable that ADAMTS13 has other, yet unidentified proteolytic activity, or competes/ interacts with glucose or currently unknown protein(s) to contribute to cognitive decline. One would expect that the pathological mechanism underlying this interaction shows similarly in the association of ADAMTS13 with related disease outcomes. A previous report of the Rotterdam Study has described an increased risk of ischaemic stroke with low ADAMTS13 activity^5^, but the link between ADAMTS13 and diabetes had not yet emerged at the time. Exploring these data further in a post-hoc analysis, we now observed patterns in the association between ADAMTS13 and risk of ischaemic stroke, similar to those with dementia in the current study (HR [95% CI] per SD decrease in ADAMTS13 for risk of ischaemic stroke in those free of (pre-)diabetes: 1.19 [1.04–1.36], versus in those with (pre-)diabetes: 0.94 [0.79–1.11]). This points towards a vascular disease related interactive mechanism, in which ADAMTS13 has a common role across diseases outcomes. While we encourage attempts for replication of our findings in other populations, we believe that current insight warrants serum glucose and diabetes history to be taken into account in future study of ADAMTS13.

Although we believe our results are reliable, there are several limitations. First, despite rigorous adjustment for known determinants of VWF and ADAMTS13, residual confounding may still exist, in particular with respect to other factors involved in hemostasis, diabetes, or possibly angiogenesis and extracellular matrix stability. Second, although follow-up for dementia was near-complete, attrition for repeated detailed cognitive assessment was substantial. Third, the association between ADAMTS13 and diabetes was first described in the same cohort as drawn from in the present analyses, and (large-scale) replication is warranted. Fourth, the Rotterdam Study population is predominantly of Caucasian descent, and levels and effects of ADAMTS13 might differ across ethnicities.

In conclusion, higher VWF and low ADAMTS13 activity are associated with accelerated cognitive decline and increased risk of dementia. However, associations with VWF are restricted to short-term risks, and do not display synergistic effects with ADAMTS13 on dementia risk. The impact of diabetes on the effect of ADAMTS13 on dementia (as well as ischaemic stroke), further emphasises the need to unravel the biological function of ADAMTS13.

## Electronic supplementary material

Supplementary Information
