# Supplementary material for: Von Willebrand factor and ADAMTS13 activity in relation to risk of dementia: a population-based study
Source: Sci Rep. 2018 Apr 3;8:5474. doi: 10.1038/s41598-018-23865-7 (PMC5882924; doi:10.1038/s41598-018-23865-7)
Supplement: Supplementary file 1 — Supplementary Information [file 41598_2018_23865_MOESM1_ESM.pdf]

Supplementary material with the article

Von Willebrand factor and ADAMTS13 activity in relation to risk of dementia: a population-based study

Frank J Wolters, Johan Boender, Paul S de Vries, Michelle A Sonneveld, Peter J Koudstaal, Moniek P de Maat, Oscar H Franco, M Kamran Ikram, Frank W Leebeek, M Arfan Ikram

Supplementary Figure S1. Associations of Von Willebrand factor (VWF) and ADAMTS13 with risk of dementia for several subgroups of interest.

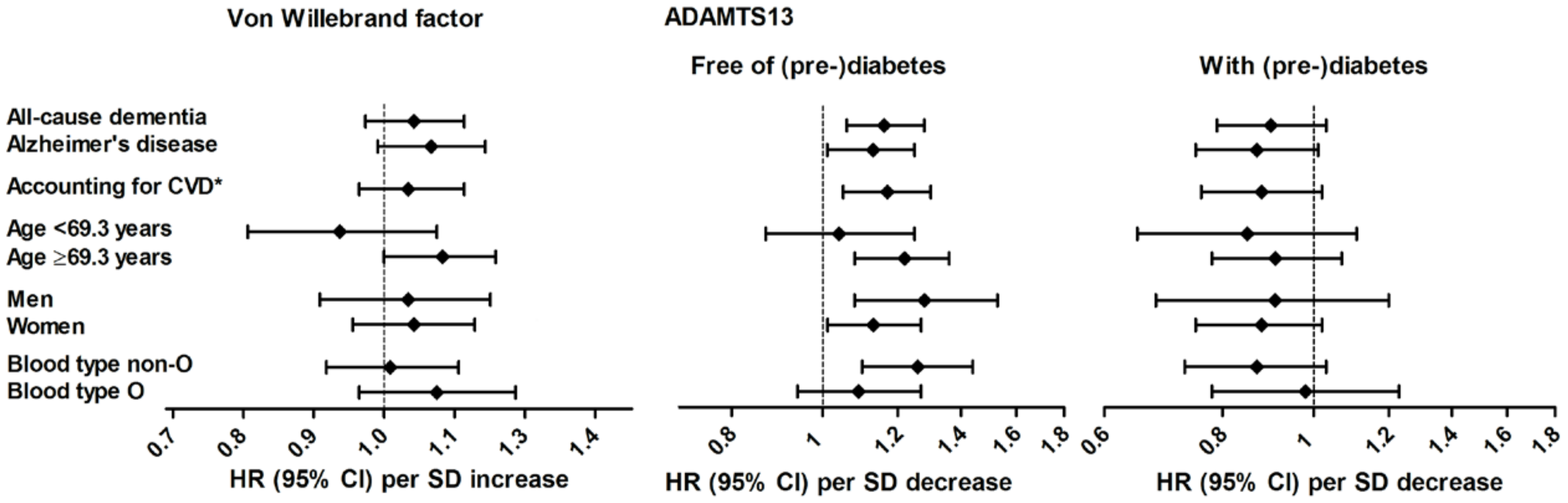

Legend: HR=hazard ratio from fully adjusted model; CI=confidence interval; SD=standard deviation
Age stratification was done by the median age of the study population.

Supplementary Table S1 Von Willebrand factor (VWF) and ADAMTS13 at baseline in relation to the prevalence of Alzheimer's disease, and all-cause dementia excluding participants with cardiovascular disease at baseline.

	**Alzheimer's disease**			**All-cause dementia excluding cardiovascular disease**		
	No (n=5970)	Yes (n=68)	P-value	No (n=5674)	Yes (n=72)	P-value
**VWF:Ag**						
Geometric mean (95% CI)	1.22 (1.20-1.23)	1.31 (1.19-1.44)	0.119	1.21 (1.20-1.22)	1.37 (1.25-1.50)	0.011
OR (95% CI) per SD increase	1.22 (0.95-1.56)		0.122	1.34 (1.06-1.69)		0.016
**ADAMTS13 activity**						
Mean (95% CI)	91.2 (90.7-91.6)	85.0 (80.9-89.0)	0.003	91.2 (90.8-91.7)	86.0 (82.1-89.9)	0.009
OR (95% CI) per SD increase	1.36 (1.03-1.78)		0.029	1.30 (1.00-1.70)		0.052
**ADAMTS13:VWF ratio**						
Geometric mean (95% CI)	73.5 (72.6-74.3)	62.4 (56.1-69.5)	0.003	73.8 (73.0-74.8)	61.1 (55.0-67.8)	0.0004
OR (95% CI) per SD decrease	1.35 (1.06-1.72)		0.016	1.43 (1.13-1.80)		0.003

n=number of participants; se=standard error; SD=standard deviation; OR=odds ratio; CI=confidence interval;
Model adjusted for age, sex, study subcohort

Supplementary Table S2 Baseline levels of Von Willebrand factor antigen and risk of dementia, depicted per increasing year of follow-up

	All-cause dementia<br>n/N	Odds ratio/ Hazard ratios (95% confidence interval)<br>Per standard deviation increase
**Cross-sectional**	85/6055	1.37 (1.06-1.77)
**Longitudinal**		
≤2 years	61/5970	1.32 (1.02-1.72)
≤3 years	111/5970	1.21 (1.00-1.48)
≤4 years	125/5970	1.15 (0.96-1.39)
≤5 years	204/5970	1.07 (0.93-1.24)
≤6 years	263/5970	1.08 (0.95-1.23)
≤7 years	322/5970	1.06 (0.95-1.19)
≤8 years	360/5970	1.08 (0.97-1.20)
≤9 years	399/5970	1.05 (0.95-1.17)
≤10 years	455/5970	1.07 (0.97-1.19)

n=number of dementia cases; N=sample at risk. Model adjusted for age, sex, study subcohort, smoking, systolic and diastolic blood pressure, antihypertensive medication, diabetes, serum cholesterol, high-density lipoprotein and triglycerides, lipid-lowering medication, creatinine, antithrombotic medication, fibrinogen, C-reactive protein, ABO blood type, and *APOE* genotype

Supplementary Table S3 Von Willebrand factor in relation to cognitive performance at baseline and during follow-up.

Cognitive test	Cross-sectional	Up till 2nd examination (4.4 years)	Up till 3rd examination (10.8 years)	Up till 4th examination (15.4 years)
**Letter-digit substitution –** *per SD*	*-0.026 (-0.051;-0.003)*	*-0.016 (-0.061;0.029)*	*0.012 (-0.016;0.039)*	*-0.001 (-0.022;0.021)*
Q2 (vs. Q1)	-0.015 (-0.079; 0.048)	-0.013 (-0.133 ;0.107)	0.017 (-0.054; 0.088)	0.017 (-0.037; 0.071)
Q3 (vs. Q1)	-0.005 (-0.071; 0.061)	0.012 (-0.110; -0.134)	0.012 (-0.060; 0.084)	-0.012 (-0.067; 0.044)
Q4 (vs. Q1)	-0.081 (-0.151; -0.011)	-0.066 (-0.192; 0.060)	0.045 (-0.031; 0.122)	0.007 (-0.052; 0.066)
**Verbal fluency** – *per SD*	*-0.050 (-0.076;-0.024)*	*-0.037 (-0.096;0.022)*	*0.020 (-0.010;0.050)*	*0.006 (-0.017;0.029)*
Q2 (vs. Q1)	-0.061 (-0.127; 0.005)	-0.032 (-0.190; 0.125)	0.003 (-0.075; 0.081)	0.022 (-0.036; 0.081)
Q3 (vs. Q1)	-0.031 (-0.100; 0.037)	-0.145 (-0.305; 0.016)	-0.021 (-0.100; 0.059)	-0.036 (-0.097; 0.024)
Q4 (vs. Q1)	-0.157 (-0.230; -0.085)	-0.069 (-0.233; 0.096)	0.059 (-0.025; 0.143)	0.021 (-0.044; 0.086)
**Stroop test** – *per SD*	*-0.031 (-0.057;-0.005)*	*-0.049 (-0.110;0.012)*	*-0.015 (-0.046;0.017)*	*-0.027 (-0.053;-0.0004)*
Q2 (vs. Q1)	-0.028 (-0.094; 0.037)	-0.024 (-0.185; 0.136)	0.032 (-0.050; 0.113)	0.008 (-0.059; 0.074)
Q3 (vs. Q1)	-0.044 (-0.112; 0.024)	-0.009 (-0.172; 0.154)	0.024 (-0.059; 0.107)	-0.027 (-0.095; 0.041)
Q4 (vs. Q1)	-0.103 (-0.175; -0.030)	-0.093 (-0.263; 0.076)	-0.007 (-0.095; 0.081)	-0.049 (-0.122; 0.023)
**G-factor** – *per SD*	*-0.047 (-0.072;-0.021)*	*-0.057 (-0.103;-0.010)*	*-0.005 (-0.030;0.019)*	*-0.013 (-0.034;0.007)*
Q2 (vs. Q1)	-0.050 (-0.113; 0.014)	-0.021 (-0.144; 0.103)	0.025 (-0.038; 0.089)	0.024 (-0.028; 0.075)
Q3 (vs. Q1)	-0.039 (-0.105; 0.027)	-0.089 (-0.214; 0.036)	-0.001 (-0.066; 0.064)	-0.033 (-0.086; 0.020)
Q4 (vs. Q1)	-0.156 (-0.227; -0.086)	-0.123 (-0.252; 0.007)	0.004 (-0.065; 0.073)	-0.024 (-0.081; 0.033)

SD=standard deviation. Results reflect the betas per standard deviation increase for baseline VWF and the VWF*follow-up time interaction (expressed per 10 years follow-up) from a fully adjusted linear mixed model including all four examinations, and restricting analyses to the first two or three assessments, respectively. Lower scores reflect worse performance for all tests.

Supplementary Table S4 Baseline ADAMTS13 in relation to the risk of dementia in the overall population: comparison of model I with model II, and stratifying between pre-diabetes and diabetes at baseline

	**Overall study population**		**Free of (pre-)diabetes**		**Pre-diabetes**		**Diabetes**	
	n_{dem}/N_{tot}	HR (95% CI)	n_{dem}/N_{tot}	HR (95% CI)	n_{dem}/N_{tot}	HR (95% CI)	n_{dem}/N_{tot}	HR (95% CI)
**Model I**								
Per quartile								
Q1 $_{<80.6\%}$	239/1492	1.09 (0.89-1.33)	190/1081	1.38 (1.07-1.77)	28/238	0.57 (0.33-0.98)	21/163	0.77 (0.44-1.35)
Q2 $_{80.6-91.2\%}$	210/1493	0.93 (0.76-1.14)	158/1088	1.10 (0.86-1.41)	31/235	0.67 (0.41-1.12)	20/154	0.75 (0.44-1.30)
Q3 $_{91.2-101.9\%}$	191/1493	0.83 (0.67-1.02)	133/1095	0.90 (0.69-1.16)	31/223	0.77 (0.46-1.28)	27/168	0.79 (0.48-1.29)
Q4 $_{>101.9\%}$	181/1492	REFERENCE	106/995	REFERENCE	31/223	REFERENCE	43/259	REFERENCE
Q1 versus Q2-4		1.19 (1.02-1.39)		1.38 (1.15-1.65)		0.73 (0.47-1.13)		0.91 (0.56-1.49)
Per SD decrease	821/5970	1.04 (0.96-1.12)	587/4259	1.13 (1.03-1.24)	121/919	0.87 (0.73-1.04)	111/744	0.97 (0.81-1.16)
**Model II**								
Per quartile								
Q1 $_{<80.6\%}$	239/1492	1.16 (0.94-1.43)	190/1081	1.51 (1.16-1.95)	28/238	0.52 (0.29-0.91)	21/163	0.87 (0.48-1.55)
Q2 $_{80.6-91.2\%}$	210/1493	1.00 (0.82-1.23)	158/1088	1.22 (0.94-1.57)	31/235	0.65 (0.38-1.11)	20/154	0.79 (0.44-1.41)
Q3 $_{91.2-101.9\%}$	191/1493	0.85 (0.69-1.04)	133/1095	0.90 (0.69-1.17)	31/223	0.75 (0.44-1.26)	27/168	0.84 (0.50-1.41)
Q4 $_{>101.9\%}$	181/1492	REFERENCE	106/995	REFERENCE	31/223	REFERENCE	43/259	REFERENCE
Q1 versus Q2-4		1.23 (1.05-1.44)		1.44 (1.20-1.73)		0.69 (0.44-1.07)		0.98 (0.59-1.64)
Per SD decrease	821/5970	1.06 (0.98-1.15)	587/4259	1.16 (1.06-1.28)	121/919	0.83 (0.68-1.01)	111/744	1.04 (0.85-1.27)

HR=hazard ratio; CI=confidence interval; n_{dem}=number of dementia cases and N_{tot}=number of individuals in group, presented for non-imputed data (missing diabetes status, n=48)

Model I: adjusted for age, sex, and study subcohort

Model II: as model I with additional adjustment for smoking, systolic and diastolic blood pressure, antihypertensive medication, serum cholesterol, HDL and triglycerides, lipid-lowering medication, body mass index, (pre-)diabetes (if applicable), creatinine, antithrombotic medication, CRP, fibrinogen, ABO blood type, and *APOE* genotype

Supplementary Table S5 Baseline ADAMTS13:VWF ratio and risk of dementia.

		Overall analysis (n=5970)		Free of (pre-)diabetes (n=4259)		With (pre-)diabetes (n=1663)	
		**Model I**	**Model II**	**Model I**	**Model II**	**Model I**	**Model II**
**ADAMTS13:VWF ratio**	n_{dem}/N_{tot}	HR, 95% CI	HR, 95% CI	HR, 95% CI	HR, 95% CI	HR, 95% CI	HR, 95% CI
Per quartile							
Q1	239/1492	1.12 (0.92-1.38)	1.20, 0.96-1.49	1.30 (1.01-1.66)	1.40 (1.10-1.82)	0.80 (0.56-1.15)	0.81 (0.54-1.22)
Q2	210/1493	0.97 (0.79-1.19)	0.98, 0.79-1.21	1.08 (0.84-1.38)	1.07 (0.83-1.39)	0.76 (0.53-1.10)	0.77 (0.52-1.13)
Q3	191/1493	1.16 (0.94-1.43)	1.21, 0.98-1.49	1.34 (1.05-1.72)	1.41 (1.10-1.82)	0.79 (0.54-1.17)	0.82 (0.55-1.22)
Q4	181/1492	REFERENCE	REFERENCE	REFERENCE	REFERENCE	REFERENCE	REFERENCE
Per SD decrease	821/5970	1.05 (0.98-1.13)	1.08 (1.00-1.17)	1.08 (0.99-1.18)	1.11 (1.01-1.23)	0.99 (0.86-1.13)	0.99 (0.86-1.17)

Model adjusted for age, sex, study subcohort, smoking, systolic and diastolic blood pressure, antihypertensive medication, diabetes, serum cholesterol, HDL and triglycerides, lipid-lowering medication, body mass index, diabetes, creatinine, antithrombotic medication, CRP, fibrinogen, and *APOE* genotype

Supplementary Table S6 Quartiles of ADAMTS13 activity in relation to change in cognitive test scores during four consecutive rounds

ADAMTS13	Free of (pre-)diabetes (n=4259)<br>β, 95% CI	Impaired fasting glucose (n=919)<br>β, 95% CI	Diabetes (n=744)<br>β, 95% CI
**G-factor**			
Q1	-0.051 (-0.117; 0.015)	-0.088 (-0.233; 0.057)	0.046 (-0.147; 0.238)
Q2	0.006 (-0.054; 0.066)	-0.021 (-0.155; 0.114)	0.002 (-0.190; 0.194)
Q3	0.009 (-0.049; 0.067)	0.057 (-0.079; 0.193)	-0.015 (-0.189; 0.158)
Q4	REFERENCE	REFERENCE	REFERENCE
Q1 versus Q2-4	-0.056 (-0.113; 0.001)	-0.098 (-0.223; 0.026)	0.050 (-0.122; 0.222)
**Letter-digit substitution**			
Q1	-0.044 (-0.114; 0.025)	0.001 (-0.144; 0.147)	0.078 (-0.118; 0.275)
Q2	0.005 (-0.059; 0.068)	-0.007 (-0.143; 0.129)	-0.075 (-0.272; 0.123)
Q3	-0.001 (-0.063; 0.060)	0.128 (-0.010; 0.265)	-0.049 (-0.228; 0.131)
Q4	REFERENCE	REFERENCE	REFERENCE
Q1 versus Q2-4	-0.046 (-0.106; 0.014)	-0.036 (-0.160; 0.088)	0.113 (-0.063; 0.288)
**Verbal fluency**			
Q1	-0.014 (-0.089; 0.062)	-0.073 (-0.254; 0.108)	0.001 (-0.195; 0.197)
Q2	0.059 (-0.010; 0.127)	0.016 (-0.151; 0.183)	-0.020 (-0.213; 0.174)
Q3	0.005 (-0.062; 0.071)	0.161 (-0.007; 0.329)	0.037 (-0.143; 0.216)
Q4	REFERENCE	REFERENCE	REFERENCE
Q1 versus Q2-4	-0.034 (-0.099; 0.031)	-0.129 (-0.284; 0.026)	-0.007 (-0.181; 0.167)
**Stroop test**			
Q1	-0.060 (-0.141; 0.021)	-0.252 (-0.476; -0.028)	0.100 (-0.164; 0.363)
Q2	-0.009 (-0.083; 0.064)	-0.130 (-0.340; 0.080)	0.047 (-0.216; 0.310)
Q3	0.004 (-0.067; 0.075)	-0.118 (-0.329; 0.092)	-0.065 (-0.306; 0.177)
Q4	REFERENCE	REFERENCE	REFERENCE
Q1 versus Q2-4	-0.059 (-0.129; 0.011)	-0.170 (-0.359; 0.019)	0.108 (-0.127; 0.343)

Model adjusted for age, sex, study subcohort, smoking, systolic and diastolic blood pressure, antihypertensive medication, serum cholesterol, HDL and triglycerides, lipid-lowering medication, body mass index, creatinine, antithrombotic medication, CRP, fibrinogen, ABO blood type, and *APOE* genotype. Estimates represent change in cognitive performance per 10 years of follow-up, relative to the highest quartile(s).

Supplementary Table S7 ADAMTS13:VWF ratio and change in cognitive test scores during the 15-year follow-up period.

	β, 95% CI
**G-factor**	
Q1	-0.084 (-0.142; -0.026)
Q2	0.003 (-0.050; 0.056)
Q3	-0.005 (-0.055; 0.045)
Q4	REFERENCE
**Letter-digit substitution**	
Q1	-0.013 (-0.074; 0.048)
Q2	-0.004 (-0.060; 0.051)
Q3	-0.003 (-0.056; 0.050)
Q4	REFERENCE
**Verbal fluency**	
Q1	-0.009 (-0.076; 0.058)
Q2	0.003 (-0.058; 0.064)
Q3	-0.025 (-0.083; 0.033)
Q4	REFERENCE
**Stroop test**	
Q1	-0.113 (-0.188; -0.038)
Q2	0.012 (-0.056; 0.080)
Q3	0.033 (-0.032; 0.098)
Q4	REFERENCE

Model adjusted for age, sex, study subcohort, smoking, systolic and diastolic blood pressure, antihypertensive medication, serum cholesterol, HDL and triglycerides, lipid-lowering medication, BMI, diabetes, creatinine, antithrombotic medication, CRP, fibrinogen, ABO blood type, and *APOE* genotype. Estimates represent change in cognitive performance per 10 years of follow-up, relative to the highest quartile.

Supplementary Table S8 ADAMTS13 activity and cognitive change, excluding participants who developed dementia during follow-up

	**Overall population (n=5970)**<br>β, 95% CI	**Excluding incident dementia (n=5149)**<br>β, 95% CI
**G-factor**		
Q1	-0.044 (-0.102; 0.014)	-0.055 (-0.107; -0.003)
Q2	0.009 (-0.044; 0.061)	-0.002 (-0.050; 0.046)
Q3	0.020 (-0.031; 0.071)	0.017 (-0.029; 0.063)
Q4	REFERENCE	REFERENCE
Q1 versus Q2-4	-0.053 (-0.103; -0.004)	-0.061 (-0.106; -0.015)
**Letter-digit substitution**		
Q1	-0.021 (-0.081; 0.039)	-0.030 (-0.076; 0.017)
Q2	-0.0002 (-0.056; 0.055)	-0.034 (-0.077; 0.009)
Q3	0.016 (-0.037; 0.070)	0.002 (-0.039; 0.044)
Q4	REFERENCE	REFERENCE
Q1 versus Q2-4	-0.027 (-0.079; 0.025)	-0.020 (-0.060; 0.020)
**Verbal fluency**		
Q1	-0.019 (-0.084; 0.047)	-0.036 (-0.099; 0.027)
Q2	0.052 (-0.008; 0.113)	0.046 (-0.012; 0.103)
Q3	0.034 (-0.025; 0.092)	0.010 (-0.046; 0.066)
Q4	REFERENCE	REFERENCE
Q1 versus Q2-4	-0.047 (-0.104; 0.010)	-0.054 (-0.108; 0.001)
**Stroop test**		
Q1	-0.074 (-0.148; 0.0001)	-0.071 (-0.137; -0.006)
Q2	-0.019 (-0.087; 0.050)	-0.028 (-0.089; 0.033)
Q3	-0.012 (-0.078; 0.054)	0.007 (-0.051; 0.065)
Q4	REFERENCE	REFERENCE
Q1 versus Q2-4	-0.064 (-0.128; -0.0002)	-0.065 (-0.122; -0.008)

Model adjusted for age, sex, study subcohort, smoking, systolic and diastolic blood pressure, antihypertensive medication, serum cholesterol, HDL and triglycerides, lipid-lowering medication, BMI, diabetes, creatinine, antithrombotic medication, CRP, fibrinogen, ABO blood type, and *APOE* genotype.
